# Microbial Glycosylation of Daidzein, Genistein and Biochanin A: Two New Glucosides of Biochanin A

**DOI:** 10.3390/molecules22010081

**Published:** 2017-01-03

**Authors:** Sandra Sordon, Jarosław Popłoński, Tomasz Tronina, Ewa Huszcza

**Affiliations:** Department of Chemistry, Wrocław University of Environmental and Life Sciences, Norwida 25, 50-375 Wrocław, Poland; jaroslaw.poplonski@up.wroc.pl; (J.P.); tomasz.tronina@up.wroc.pl (T.T.); ewa.huszcza@up.wroc.pl (E.H.)

**Keywords:** isoflavones, daidzein, genistein, biochanin A, microbial glycosylation, fungi

## Abstract

Biotransformation of daidzein, genistein and biochanin A by three selected filamentous fungi was investigated. As a result of biotransformations, six glycosylation products were obtained. Fungus *Beauveria bassiana* converted all tested isoflavones to 4″-*O*-methyl-7-*O*-glucosyl derivatives, whereas *Absidia coerulea* and *Absidia glauca* were able to transform genistein and biochanin A to genistin and sissotrin, respectively. In the culture of *Absidia coerulea*, in addition to the sissotrin, the product of glucosylation at position 5 was formed. Two of the obtained compounds have not been published so far: 4″-*O*-methyl-7-*O*-glucosyl biochanin A and 5-*O-*glucosyl biochanin A (isosissotrin). Biotransformation products were obtained with 22%–40% isolated yield.

## 1. Introduction

Isoflavones are the most well-known phytoestrogens [1]. They are secondary metabolites of the very restricted distribution in the plant kingdom, found exclusively in legumes (*Leguminosae* family). The major dietary sources of this most common form of phytoestrogens are soybeans, chickpeas and lentils [2]. Isoflavones have been implicated in the prevention of hormone-dependent diseases, such as: cancers (including breast, prostate and colon carcinoma) [3,4,5,6], cardiovascular disorders [7,8], bone health problems [9] and postmenopausal symptoms [10,11]. Therefore, there is considerable interest in the use of isoflavones in the prevention of estrogen-related cancers and certain diseases caused by estrogen deficiency.

Genistein and daidzein, the major isoflavones present in legumes, exist predominantly in the glycoside form, i.e., genistin and daidzin or 4′-methoxy derivatives, i.e., biochanin A and formononetin, respectively [2].

Due to better solubility, greater stability and functionality compared to aglycones, glycosylated forms of flavonoids have recently been gaining increasing attention. Biotransformations of flavonoids is a useful tool to obtain their glycosylated derivatives. Application of microorganisms as biocatalysts allows us to obtain these compounds in sufficient amounts for research, e.g., the effect of a glycoside group on compound properties and for further application as ingredients of dietary supplements and pharmaceuticals [12].

The sugar moiety of flavonoids was proposed to be the major determinant of their absorption in humans [13]. It is generally believed that flavonoid glycosides are converted to the corresponding aglycones by the intestinal microflora and/or by the intestinal glucosidases, and as such absorbed from the small intestine [14]. Many studies have shown that flavonoid glycosides are poorly absorbed compared with their aglycones [13,15,16]. This phenomenon was partially revised recently as a result of studies on the bioavailability of flavonol quercetin. It turned out that quercetin-3-*O*-β-d-glucoside has higher bioavailability than its aglycone in humans, what suggests that conjugation with glucose would enhance quercetin absorption in the small intestine [17,18,19]. However, in the case of isoflavones, there are many evidences that aglycones have more biological effects than related glycosides, due to faster absorption in a greater amount [20,21]. These studies were focused on the most common isoflavone 7-*O*-glucosides, genistin and daidzin.

In this paper we described fungal biotransformation of three isoflavones: daidzein, genistein and biochanin A. We found that substituents in the B-ring of tested flavonoids showed the impact on the regioselectivity of sugar moiety coupling.

## 2. Results and Discussion

Our previous research on microbial metabolism of flavonoids revealed that the fungal strains *Beauveria bassiana* AM 278, *Absidia glauca* AM 177 and *Absidia coerulea* AM 93 are able to attach sugar moiety to the chalcones and flavanones [22,23,24]. Therefore, biotransformation of daidzein, genistein and biochanin A, the aglycones of the most commonly occurring isoflavones, using those microorganisms was investigated.

**Figure 1:**
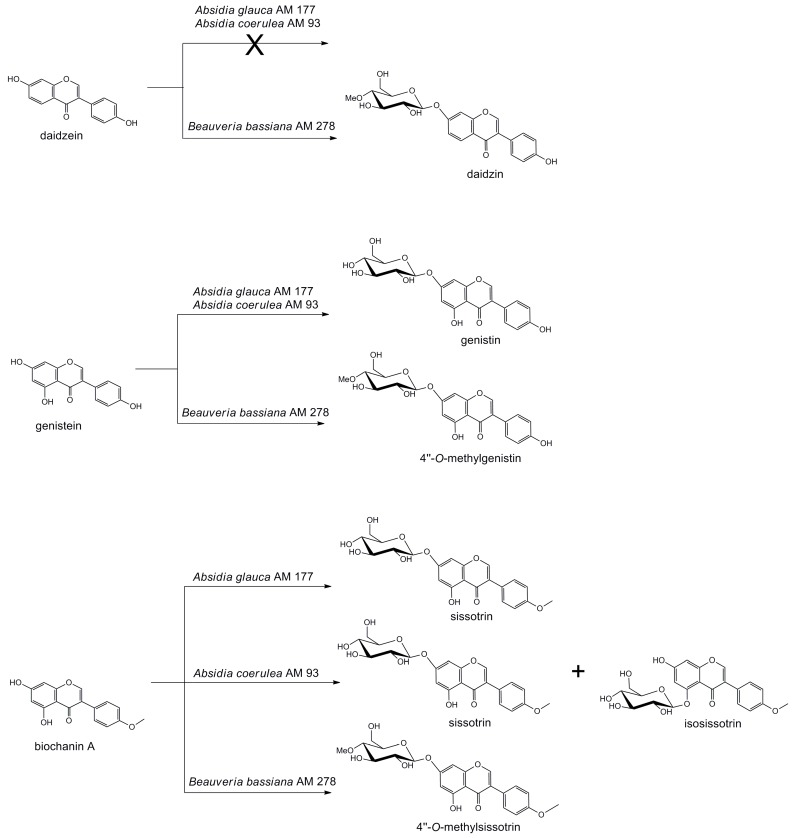
Transformation of isoflavonoids by selected fungi.

Screening tests have shown that genistein and biochanin A were transformed by all of the tested strains, while daidzein was metabolized only by the fungus *Beauveria bassiana*. Experiments conducted on a larger scale enabled us to determine the chemical structure of the resulting products and their isolated yields (Figure 1).

Daidzein was converted to 4″-*O*-methyldaidzin by *Beauveria bassiana* with a yield of 33.3% (33.8 mg). This compound was earlier identified only in a soybean broth fermented by *Paecilomyces militaris* [25].

Genistein was metabolized by *Absidia glauca* and *Absidia coerulea* to genistin with a yield of 21.9% (21.0 mg) and 12.5% (12.0 mg), respectively. Glucosylation at C-7 position of genistein was also observed for solvent-tolerant bacterium *Staphylococcus saprophyticus* CQ16 [26], as well as for plant cultured cells of *Eucalyptus perriniana* [27]. As it was expected based on our previous studies [22,23,24], fungus *Beauveria bassiana* transformed genistein to the typical for this species derivative-4″-*O*-methylgenistin (27.0 mg, 27.2%).

Incubation of biochanin A with *Beauveria bassiana* led to the new metabolite 4″-*O*-methylbiochanin A (29.9 mg, 30.8%). Seven proton and six carbon signals were observed in the regions from 3.21 to 5.03 ppm in the ^1^H-NMR and from 60.9 to 101.5 ppm in the ^13^C-NMR spectra. HSQC, HMBC and COSY spectra proved that the signals were corresponding to a glucose molecule. The methyl carbon (C-4″OCH_3_) resonating at δ 60.9 showed a one-bond correlation with three protons at δ 3.60 on HMQC spectrum. These protons also showed three-bond correlation with carbon at δ 80.5 (C4″) on HMBC spectrum. Discussed metabolite had the molecular formula (C_23_H_24_O_10_-H)^−^ as indicated by HR-ESIMS of the [M − H]-ion peak at *m*/*z* 459.1297 (calcd. 459.1296).

When biochanin A was transformed by *Absidia glauca* as a bioreagent, the sissotrin was observed as the only product (38.0 mg, 40.3%). Glucosylation of biochanin A at C-7 position was also performed by *Absidia coerulea*, however with a much lower yield (4.5 mg, 4.6%). This phenomenon can be attributed to concurrent formation of the second metabolite, which was recognized as 5-*O*-β-glucosylbiochanin A. The yield of this positional isomer of sissotrin, named isosissotrin by us, was 23.2% (22.5 mg). The composition of the products mixture was determined based on the NMR spectra analyses.

The NMR spectra of products obtained by biotransformation by *Absidia coerulea* AM 93 showed characteristic doubled signals (Figures S1–S12, Supplementary Materials). HMQC, HMBC and COSY experiments allowed us to establish unambiguously the flavonoid glycosides structure. The presence of doubled signals typical for a glucose moiety suggested the formation of two glucosylation products. In the ^1^H-NMR and ^13^C-NMR spectrum, doubled six proton and doubled six carbon signals corresponding to a glucose moiety structure were observed. The proton signal of H-1″ of glucosylation product at C-5 position was overlapped with the artifact peak (likely of water) (Figures S1, S3, S5, S7, S8 and S10 Supplementary Materials).

Correlations observed in the HMBC spectrum permitted the assignment of the H-6 and H-8 protons of the two flavonoid products (Figure S9, Supplementary Materials). The aromatic A-ring protons H-6 appearing as doublets at δ 6.83 (*J* = 2.2 Hz) and 6.51 (*J* = 2.2 Hz) resonated with three carbons (C-10: 110.3 ppm; C-5: 160.6 ppm; C-7: 164.7 ppm) and (C-10: 108.0 ppm; C-5: 163.5 ppm; C-7: 164.8 ppm) respectively. In turn, the other A-ring protons H-8 appearing as doublets at δ 6.58 (*J* = 2.2 Hz) and 6.70 (*J* = 2.2 Hz) resonated with four carbons (C-6: 104.5 ppm; C-10: 110.3 ppm; C-9: 160.9 ppm; C-7: 164.7 ppm) and (C-6: 101.2 ppm; C-10: 108.0 ppm; C-9: 159.2 ppm; C-7: 164.8 ppm), respectively.

The HMBC correlation between H-1″ and C-7 and between H-1″ and C-5 confirmed the location of sugar molecules. Noteworthy is that no significant signal shifts of both protons and carbons of the ring A of this product were observed, thus providing further confirmation of glucosyl moiety attachment. The glucose moieties were connected by 1,7-glycosidic linkages in sissotrin and by a 1,5-glycosidic linkage in isosissotrin (Figure S10, Supplementary Materials).

The products showed the [M − M]-ion peak at *m*/*z* 445.1151 (calcd. 445.1140) in HR-ESIMS. The exact molecular mass was consistent with a molecular formula of C_22_H_22_O_10_ and confirmed the formation of two positional isomers (Figure S13, Supplementary Materials).

Lewis et al. described the chemical synthesis of daidzin, genistin and sissotrin by the phase transfer catalyzed reaction between isoflavone aglycones of obtained products and 1-bromo-2,3,4,6-tetra-*O*-acetyl-α-glucopyranose in the presence of tetrabutylammonium bromide as a catalyst [28]. This method has some disadvantages: the catalyst used in the reaction is harmful for the environment and the protection of hydroxyl groups that are not meant to conjugate with saccharide is necessary. Moreover, chemical synthesis does not solve the synthetic challenges of flavonoid 5-*O*-glucosides [29].

The use of filamentous fungi as a biocatalyst is the relevant method for obtaining isoflavonoid glucosides in simple and cheap one-step biotransformation process. Furthermore, according to the European Union Law, the products obtained by biotransformation are classified as natural compounds (EU Directive 88/388/EEC).

Summarizing, the course of the biotransformation of isoflavones by three selected filamentous fungi known from their ability to *O*-glycosylation was investigated. High regioselectivity maintaining low substrate specificity was observed in biotransformation conducted by *Beauveria bassiana*, which is typical for this species and has been shown in previous studies [22,23,24,30,31,32]. Whilst, the substrate specificity of enzymes from *Absidia* genus is higher. In the presented studies it was observed that isoflavones without a hydroxyl group at the C-5 position do not undergo biotransformation by *Absidia* species. In addition, presence of the methoxy group at C-4′ position change the regioselectivity of biotransformations by *Absidia coerulea*, which may be associated with different substrate orientation in the catalytic pocket of the enzyme or with different enzyme catalyzing conversion of this substrates. Fungus *Absidia coerulea* glucosylates biochanin A mainly at the 5-OH position, whereas *Absidia glauca* metabolizes biochanin A only to one glucosylation product at the 7-OH position.

As we expected, the microorganisms used in the studies carried out conjugation of isoflavones with the sugar molecule. Daidzin and genistin occur naturally in relatively large amounts; however, sissotrin, isolated mainly from red clover, occurs in amounts more than 10-fold less than its aglycone form biochanin A [33]. Therefore, our biotechnological methods of obtaining sissotrin as well as of isosissotrin may have practical significance. The fungal transformations presented in this article turned out to be a useful tool for obtaining new isoflavone glucosides as well as these, which naturally occur in small quantities. Due to relatively high yields (compared to chemical methods) the filamentous fungi biocatalysis can be used as a method of choice for bioavailability studies. There are many studies on bioavailability of genistein and its glucoside genistin and also daidzein and its glucoside daidzin, although absorption of isoflavonoid glycosides and their respective aglycones that form in humans is a subject of controversy [34]. Although there are reports in the literature on bioavailability of biochanin A [35,36,37], there is no data about biochanin A glucoside bioavailability, possibly due to the rarer occurrence of sissotrin in nature. Described biotransformation of biochanin A is the method that allows us to obtain a glucoside in an amount sufficient for the research concerning the influence of glucose moiety on biological activity.

## 3. Materials and Methods

### 3.1. Compounds

Biochanin A (5,7-dihydroxy-4′-methoxyisoflavone) was purchased from Sigma-Aldrich (St. Louis, MO, USA). Daidzein (4′,7-dihydroxyisoflavone) and genistein (4′,5,7-trihydroxyisoflavone) were isolated from soy extract. Flavonoid glycosides included in the dry extract were hydrolyzed and the aglycones of daidzein and genistein were isolated from the reaction mixture according to the method described by Utkina et al. [38] and were of high purity (>98% by HPLC).

*Daidzein*: ^1^H-NMR (600 MHz, DMSO-*d*_6_) δ: 6.80 (2H, m, H-3′, H-5′), 6.85 (1H, d, *J* = 2.2 Hz, H-8), 6.93 (1H, dd, *J* = 8.7, 2.2 Hz, H-6), 7.37 (2H, m, H-2′, H-6′), 7.96 (1H, d, *J* = 8.7, H-5), 8.29 (1H, s, H-2), 9.51 (1H, s, 4′-OH), 10.77 (1H, s, 7-OH); ^13^C-NMR (150 MHz, DMSO-*d*_6_) δ: 102.8 (C-8), 114.1 (C-6), 116.6 (C-10), 115.1 (C-3′, C-5′), 122.5 (C-1′), 123.5 (C-3), 127.3 (C-5), 130.1 (C-2′, C-6′), 152.8 (C-2), 157.2 (C-4′), 157.4 (C-9), 162.5 (C-7), 174.7 (C-4). 

*Genistein*: ^1^H-NMR (600 MHz, DMSO-*d*_6_) δ: 6.26 (1H, d, *J* = 2.1 Hz, H-6), 6.42 (1H, d, *J* = 2.1 Hz, H-8), 6.82 (2H, m, H-3′, H-5′), 7.37 (2H, m, H-2′, H-6′), 8.32 (1H, s, H-2), 9.65 (1H, s, 4′-OH), 11.04 (1H, s, 7-OH), 12.94 (1H, s, 5-OH); ^13^C-NMR (150 MHz, DMSO-*d*_6_) δ: 94.2 (C-8), 99.5 (C-6), 104.9 (C-10), 115.5 (C-3′, C-5′), 121.4 (C-1′), 122.7 (C-3), 130.6 (C-2′, C-6′), 154.5 (C-2), 157.9 (C-4′), 158.0 (C-9), 162.4 (C-5), 164.9 (C-7), 180.8 (C-4).

^1^H- and ^13^C-NMR data were found to be identical to that of daidzein and genistein reported in the literature [39,40].

### 3.2. Biotransformation

#### 3.2.1. Microorganisms

The microorganisms used in this work, i.e., *Beauveria bassiana* AM 278, *Absidia glauca* AM 177 and *Absidia coerulea* AM 93, were obtained from the collection of the Department of Biology and Pharmaceutical Botany, Medical University of Wrocław, Poland.

#### 3.2.2. Screening Procedure

All fermentation experiments were carried out in Sabouraud medium (d-glucose 30 g and peptone 10 g per liter of distilled water) on rotary shakers (130 rpm, 6.5 amplitude) at 28 °C. Agar slant cultures were used to obtain the inoculation culture. Portions of 0.5 mL of the 3-day inoculum were transferred to 100 mL Erlenmeyer flasks, each containing 30 mL of the medium. After cultivation, 5 mg of a substrate dissolved in 0.5 mL of dimethyl sulfoxide (DMSO) was added to the grown culture and the reaction mixture was incubated for 7 days. After incubation the cultures were acidified with 1 M HCl to pH around 5 and extracted with ethyl acetate (3 × 15 mL). The extracts were evaporated, and the residue dissolved in methanol and analyzed by TLC and HPLC. All experiments were performed with appropriate controls (the substrate in a sterile growth medium and incubation with no substrate).

#### 3.2.3. Preparative (Large) Scale Biotransformation

Portions of 1.5 mL of the 3-day inoculation culture were transferred to 300 mL Erlenmeyer flasks, each containing 100 mL of the medium. In the preparative (large scale) biotransformation, a total of 60 mg of each substrate dissolved in 6 mL of DMSO and was equally distributed among four flasks with 4-day fungal cultures to give a final concentration of 150 mg/L. After 10 days of incubation, the cultures were acidified with 1 M HCl to pH around 5 and three time extracted with the same volume of ethyl acetate. The other procedures and culture conditions were the same as those of the screening experiments. The collected organic phase was dried over anhydrous MgSO_4_, the solvent was filtered, evaporated in a vacuum and analyzed using TLC and HPLC. The biotransformation products were separated by column chromatography on silica gel 60 (230–400 mesh, Merck, Darmstadt, Germany) using chloroform: methanol (3:1 *v*/*v*) as eluent. TLC was carried out with Merck silica gel 60, F_254_ (0.2 mm thick) plates using the same eluents. Products were detected by inspecting plates under UV irradiation. *R*_f_: 0.53 (genistin); 0.64 (sissotrin, isosissotrin); 0.77 (4″-*O*-methyldaidzin); 0.79 (4″-*O*-methylgenistin); 0.84 (4″-*O*-methylsissotrin); 0.85 (genistein); 0.87 (daidzein); 0.89 (biochanin A).

### 3.3. Analysis of Products with High Performance Liquid Chromatography (HPLC)

HPLC was carried out on a Waters 2695 Alliance instrument with the photodiode array detector Waters 2996 (detection from 220 to 500 nm wavelength) using the analytical HPLC column Agilent C-18 ZORBAX Eclipse XDB 5 μm (46 mm × 250 mm) at the flow rate of 1 mL/min and injection volume 10 µL. A linear solvent gradient composed of 0.05% formic acid in water (A) and methanol containing 0.05% formic acid (B) was used. Chromatographic separation was achieved using the isocratic elution of 50% A and 50% B for 2 min, then linear gradient of B from 50% to 95% for 10 min and isocratic elution of 95% B for 2 min. RT (min): 3.79 (4″-*O*-methyldaidzin); 3.90 (genistin); 5.03 (4″-*O*-methylgenistin); 7.87 (daidzein); 8.51 (sissotrin); 8.82 (isosissotrin); 9.14 (genistein); 9.22 (4″-*O*-methylsissotrin); 12.15 (biochanin A).

### 3.4. Identification of the Products

Purified products were identified by NMR spectra analysis. ^1^H-NMR, ^13^C-NMR, ^1^H-^1^H-NMR (COSY) and ^1^H-^13^C-NMR (HSQC, HMBC) were recorded on a DRX Bruker Avance TM 600 (600 MHz) instrument. Negative-ion HR-ESIMS spectra were measured on a Bruker ESI-TOF Mass Spectrometer micrOTOF-Q. The direct infusion of ESI-MS parameters: The mass spectrometer was operated in negative ion mode with the potential between the spray needle and the orifice 4, 5 kV, nebulizer pressure of 0.4 bar, and a drying gas flow rate of 4 L/min at 200 °C. The sample flow rate was 180 μL/min. Ionization mass spectra were collected at the ranges *m*/*z* 150–3000. The instrument was calibrated with an Agilent electrospray calibration solution (ESI-L low concentration Tuning Mix-Agilent Technologies, Agilent Product Number: G1969-85000) that was diluted with acetonitrile.

### 3.5. Obtained Biotransformation Products

*7-O-β-d-4″-O-Methyl-glucopyranosyl-4′-hydroxyisoflavone* (4″-*O*-methyldaidzin), (biocatalyst: *Beauveria bassiana* AM 278, yield: 33.3%): ^1^H-NMR (600 MHz, DMSO-*d*_6_) δ: 3.06 (1H, m, H-4″), 3.31 (1H, m, H-2″), 3.45 (1H, m, H-3″), 3.47 (3H, s, 4″-OCH_3_) 3.50 (1H, m, Ha-6″), 3.52 (1H, m, H-5″), 3.65 (1H, m, Hb-6″), 4.75 (1H, m, 6″-OH), 5.13 (1H, d, *J* = 7.8 Hz, H-1″), 5.32 (1H, d, *J* = 5.5 Hz, 3″-OH), 5.52 (1H, d, *J* = 5.3 Hz, 2″-OH), 6.82 (2H, m, H-3′, H-5′), 7.14 (1H, dd, *J* = 2.3; 8.9 Hz, H-6), 7.22 (1H, d, *J* = 2.3 Hz, H-8), 7.40 (2H, m, H-2′, H-6′), 8.05 (1H, d, *J* = 8.9 Hz, H-5), 8.38 (1H, s, H-2), 9.56 (1H, s, 4′-OH); ^13^C-NMR (150 MHz, DMSO-*d*_6_) δ: 60.2 (C-6″), 73.3 (C-2″), 75.7 (C-5″), 59.7 (C-4″OCH_3_), 76.2 (C-3″), 78.9 (C-4″), 99.6 (C-1″), 103.3 (C-8), 115.0 (C-3′, 5′), 115.5 (C-6), 118.5 (C-10), 122.3 (C-1′), 123.7 (C-3), 127.0 (C-5), 130.1 (C-2′, 6′), 153.4 (C-2), 157.0 (C-9), 157.3 (C-4′), 161.3 (C-7), 174.8 (C-4). ^1^H- and ^13^C-NMR data were found to be identical to that reported in the literature [25].

*7-O-β-d-Glucopyranosyl-5,4′-dihydroxyisoflavone* (genistin), (biocatalysts: *Absidia coerulea* AM 93, yield: 12.5%; *Absidia glauca* AM 177, yield: 21.9%): ^1^H-NMR (600 MHz, DMSO-*d*_6_) δ: 3.17 (1H, m, H-4″), 3.26 (1H, m, H-2″), 3.30 (1H, m, H-3″), 3.44 (1H, m, H-5″), 3.47 (1H, m, Ha-6″), 3.71 (1H, m, Hb-6″), 4.61 (1H, m, 6″-OH), 5.06 (1H, d, *J* = 7.5 Hz, H-1″), 5.08 (1H, m, 4″-OH), 5.15 (1H, m, 3″-OH), 5.41 (1H, m, 2″-OH), 6.47 (1H, d, *J* = 2.2 Hz, H-6), 6.72 (1H, d, *J* = 2.2 Hz, H-8), 6.83 (2H, m, H-3′, H-5′), 7.40 (2H, m, H-2′, H-6′), 8.43 (1H, s, H-2), 9.63 (1H, s, 4′-OH), 12.94 (1H, s, 5-OH); ^13^C-NMR (150 MHz, DMSO-*d*_6_) δ: 60.6 (C-6″), 73.1 (C-2″), 76.4 (C3″), 69.6 (C-4″), 77.2 (C-5″), 94.5 (C-8), 99.6 (C-6), 99.8 (C-1″), 106.1 (C-10), 115.1 (C-3′, 5′), 121.0 (C-1′), 122.6 (C-3), 130.2 (C-2′, 6′), 154.6 (C-2), 157.2 (C-9), 157.5 (C-4′), 161.6 (C-5), 163.0 (C-7), 180.5 (C-4). ^1^H- and ^13^C-NMR data were found to be identical to that reported in the literature [41,42].

*7-O-β-d-4″-O-Methyl-glucopyranosyl-5,4′-dihydroxyisoflavone* (4″-*O*-methylgenistin) (biocatalyst: *Beauveria bassiana* AM 278, yield: 27.2%): ^1^H-NMR (600 MHz, DMSO-*d*_6_) δ: 3.0 (1H, m, H-4″), 3.27 (1H, m, H-2″), 3.44 (1H, m, H-3″), 3.46 (3H, s, 4″-OCH_3_) 3.49 (1H, m, Ha-6″), 3.52 (1H, m, H-5″), 3.63 (1H, m, Hb-6″), 4.73 (1H, m, 6″-OH), 5.09 (1H, d, *J* = 7.8 Hz, H-1″), 5.30 (1H, d, *J* = 5.5 Hz, 3″-OH), 5.48 (1H, d, *J* = 5.2 Hz, 2″-OH), 6.47 (1H, d, *J* = 2.2 Hz, H-6), 6.71 (1H, d, *J* = 2.2 Hz, H-8), 6.83 (2H, m, H-3′, H-5′), 7.40 (2H, m, H-2′, H-6′), 8.43 (1H, s, H-2), 9.62 (1H, s, 4′-OH), 12.94 (1H, s, 5-OH); ^13^C-NMR (150 MHz, DMSO-*d*_6_) δ: 59.7 (C-4″-OCH_3_), 60.2 (C-6″), 73.2 (C-2″), 75.7 (C-5″), 76.1 (C3″), 78.9 (C-4″), 94.5 (C-8), 99.4 (C-1″), 99.5 (C-6), 106.1 (C-10), 115.1 (C-3′, 5′), 121.0 (C-1′), 122.6 (C-3), 130.2 (C-2′, 6′), 154.6 (C-2), 157.2 (C-9), 157.5 (C-4′), 161.7 (C-5), 162.9 (C-7), 180.5 (C-4). ^1^H- and ^13^C-NMR data were found to be identical to that reported in the literature [25,30].

*7-O-β-d-Glucopyranosyl-5-hydroxy-4′-methoxyisoflavone* (sissotrin), (biocatalysts: *Absidia coerulea* AM 93, yield: 4.6%; *Absidia glauca* AM 177, yield: 40.3%): ^1^H-NMR (600 MHz, CD_3_OD) δ: 3.44–3.48 (1H, m, H-4″), 3.47–3.51 (1H, m, H-2″), 3.71–3.74 (1H, m, Ha-6″), 3.83 (3H, s, 4′-OCH_3_), 3.90–3.93 (1H, m, Hb-6″), 5.05 (1H, m, H-1″), 6.51 (1H, d, *J* = 2.2 Hz, H-6), 6.70 (1H, d, *J* = 2.2 Hz, H-8), 6.98 (2H, pd, *J* = 8.7 Hz, H-3′, H-5′), 7.48 (2H, pd, *J* = 8.7 Hz, H-2′, H-6′), 8.16 (1H, s, H-2); ^13^C-NMR (150 MHz, CD_3_OD) δ: 55.8 (C-4′OCH_3_), 62.4 (C-6″), 71.2 (C-4″), 74.7 (C-2″), 77.8 (C-3″), 78.4 (C5″), 95.9 (C-8), 101.2 (C-6), 101.6 (C-1″), 108.0 (C-10), 114.9 (C-3′, C-5′), 124.3 (C-1′), 124.8 (C-3), 131.3 (C-2′, C-6′), 155.5 (C-2), 159.2 (C-9), 161.3 (C-4′), 163.5 (C-5), 164.8 (C-7), 182.4 (C-4). ^1^H- and ^13^C-NMR data were found to be identical to that reported in the literature [43]. HRESI-MS [M − H]^−^ (calculated/found) (*m*/*z* 445.1140/445.1151).

*5-O-β-d-Glucopyranosyl-7-hydroxy-4′-methoxyisoflavone* (isosissotrin), (biocatalyst: *Absidia coerulea* AM 93, yield: 23.2%): ^1^H-NMR (600 MHz, CD_3_OD) δ: 3.40–3.44 (1H, m, H-4″), 3.41–3.51 (2H, m, H-3″, H-5″), 3.60–3.68 (1H, m, H-2″), 3.74-3.77 (1H, m, Ha-6″), 3.82 (3H, s, 4′-OCH_3_), 3.93–3.96 (1H, m, H-6b″), 4.86 (1H, signal obscured by water, H-1″), 6.58 (1H, d, *J* = 2.2 Hz, H-8), 6.83 (1H, d, *J* = 2.2 Hz, H-6), 6.95 (2H, pd, *J* = 8.7 Hz, H-3′, H-5′), 7.42 (2H, pd, *J* = 8.7 Hz, H-2′, H-6′), 8.02 (1H, s, H-2); ^13^C-NMR (150 MHz, CD_3_OD) δ: 55.7 (C-4′OCH_3_), 62.5 (C-6″), 71.2 (C-4″), 74.7 (C-2″), 77.3 (C-3″), 78.6 (C-5″), 98.9 (C-8), 104.5 (C-6), 104.9 (C-1″), 110.3 (C-10), 114.7 (C-3′, C-5′), 125.5 (C-1′), 126.7 (C-3), 131.6 (C-2′, C-6′), 153.3 (C-2), 160.6 (C-5), 160.9 (C-9), 161.1 (C-4′), 164.7 (C-7), 178.1 (C-4). HRESI-MS [M − H]^−^ (calculated/found) (*m*/*z* 445.1140/445.1151).

*7-O-β-d-4″-O-Methyl-glucopyranosyl-5-hydroxy-4′-methoxyisoflavone* (4″-*O*-methylsissotrin), (biocatalyst: *Beauveria bassiana* AM 278, yield: 30.8%): ^1^H-NMR (600 MHz, CD_3_OD) δ: 3.21 (1H, m, H-4″), 3.46–3.53 (2H, m, H-2″, H-5″), 3.60 (1H, m, H-3″), 3.60 (3H, s, 4″-OCH_3_), 3.72 (1H, m, Hb-6″), 3.83 (3H, s, 4′-OCH_3_), 3.87 (1H, m, Ha-6″), 5.03 (1H, d, *J* = 7.7 Hz, H-1″), 6.52 (1H, m, H-6), 6.70 (1H, m, H-8), 6.99 (2H, m, H-3′, H-5′), 7.49 (2H, m, H-2′, H-6′), 8.17 (1H, s, H-2); ^13^C-NMR (150 MHz, CD_3_OD) δ: 55.8 (C-4′OCH_3_), 60.9 (C-4″OCH_3_), 62.0 (C-6″), 74.8 (C-2″), 77.4 (C-5″), 77.9 (C3″), 80.5 (C-4″), 95.9 (C-8), 101.1 (C-6), 101.5 (C-1″), 108.1 (C-10), 115.0 (C-3′, 5′), 124.4 (C-1′), 124.9 (C-3), 131.4 (C-2′, 6′), 155.5 (C-2), 157.3 (C-9), 161.4 (C-4′), 163.6 (C-5), 164.8 (C-7), 182.4 (C-4). HRESI-MS [M − H]^−^ (calculated/found) (*m*/*z* 459.1296/459.1297).

## 4. Conclusions

The aim of this paper was to develop a simple and efficient method for the preparation isoflavonoid glycosides, which can serve as analytical standards or model compounds for various biological tests.

All the microorganisms that we used performed conjugation of isoflavones with the sugar molecule. Fungi *Absidia glauca* AM 177 and *Absidia coerulea* AM 93 attached glucose, while the fungus *Beauveria bassiana* AM 278 4-*O*-methylglucose. With one exception, the preferred site of glycosylation was the C-7 hydroxyl group of substrates. Thus, as a result of glucosylation of genistein and biochanin A, we obtained genistin and sissotrin, respectively. In the culture of fungi *Absidia coerulea,* biochanin A was converted to a new derivative with glucose residue at the C-5 position. To our best knowledge, this compound has not been published so far. The product of the biotransformation of biochanin A by *Beauveria bassiana* AM 278 is also reported for the first time.

## Supplementary Materials

Supplementary materials can be accessed at: http://www.mdpi.com/1420-3049/22/1/81/s1.
