# Supplementary material for: Microbial Glycosylation of Daidzein, Genistein and Biochanin A: Two New Glucosides of Biochanin A
Source: Molecules. 2017 Jan 3;22(1):81. doi: 10.3390/molecules22010081 (PMC6155839; doi:10.3390/molecules22010081)
Supplement: Supplementary file 1 [file molecules-22-00081-s001.pdf]

Supplementary Materials: Microbial Glycosylation of Daidzein, Genistein and Biochanin A. Two New Glucosides of Biochanin A

Sandra Sordon, Jarosław Popłoński, Tomasz Tronina and Ewa Huszcza

List of contents:

1. NMR spectra of compounds obtained by biotransformation with the use of *Absidia coerulea* AM 93 strain indicating the mixture of products: 7-*O*-β-D-glucopyranosyl-5-hydroxy-4′-methoxyisoflavone (sissotrin) and 5-*O*-β-D-glucopyranosyl-7-hydroxy-4′-methoxyisoflavone (isosissotrin).
   Figures S1–S2: ^{1}H-NMR (600 MHz, CD_3OD) spectra.
   Figure S3: COSY (150 MHz, CD_3OD) spectra.
   Figure S4: ^{13}C-NMR (150 MHz, CD_3OD) spectra.
   Figures S5–S7: HSQC (150 MHz, CD_3OD) spectra.
   Figures S8–S11: HMBC (150 MHz, CD_3OD) spectra.
   Figure S12: ^{13}C-NMR (150 MHz, CD_3OD) superimposed spectra of sissotrin and mixture of sissotrin and isosissotrin.
2. Negative-ion HR-ESIMS spectra of mixture of 7-*O*-β-D-glucopyranosyl-5-hydroxy-4′-methoxyisoflavone (sissotrin) and 5-*O*-β-D-glucopyranosyl-7-hydroxy-4′-methoxyisoflavone (isosissotrin) obtained by biotransformation by *Absidia coerulea* AM 93.
   Figure S13: HR-ESIMS spectrum.

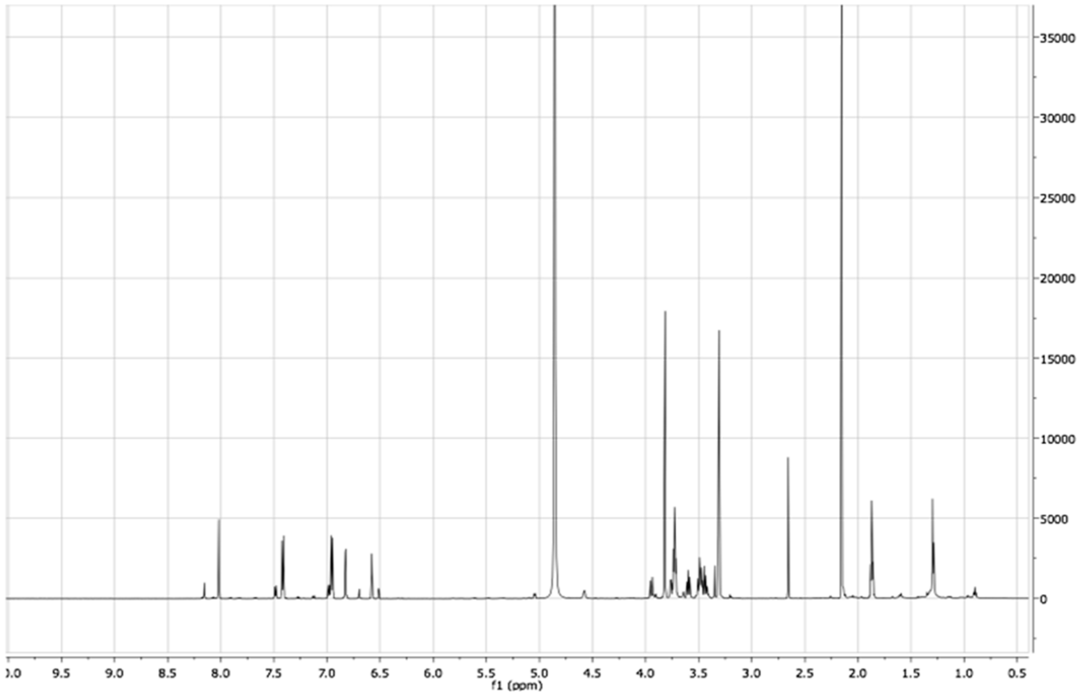

Figure S1. ^{1}H-NMR (600 MHz, CD_3OD) spectra of mixture of sissotrin and isosissotrin obtained by biotransformation by *Absidia coerulea* AM 93.

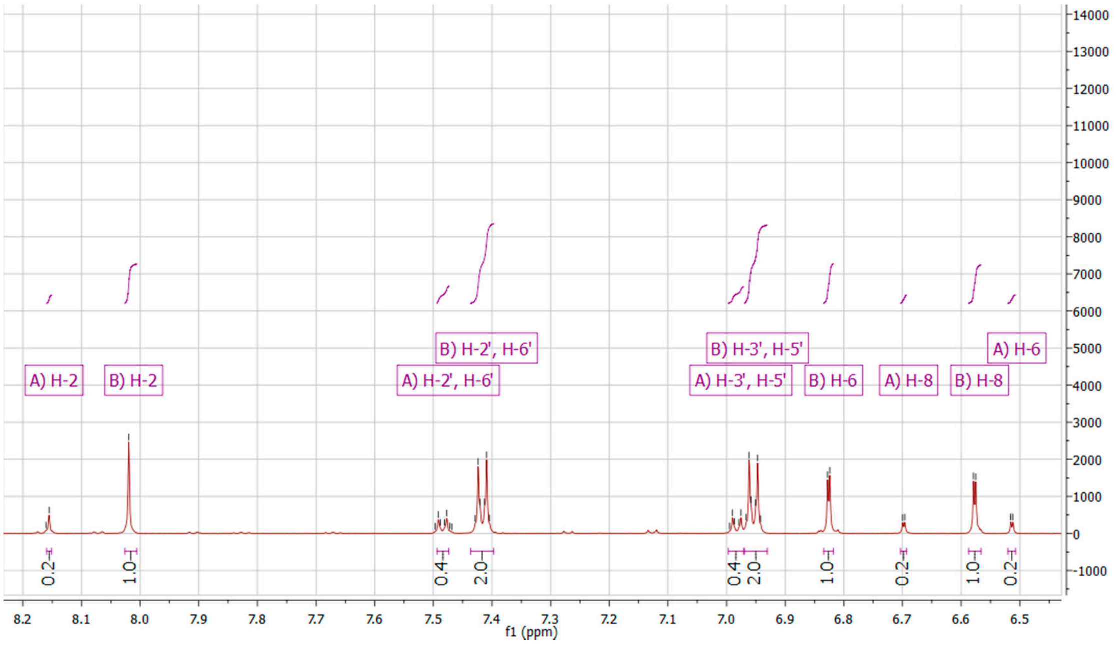

Figure S2. ^{1}H-NMR (600 MHz, CD_3OD) spectra of the aromatic region of sissotrin (**A**) and isossisotrin (**B**) mixture obtained by biotransformation by *Absidia coerulea* AM 93.

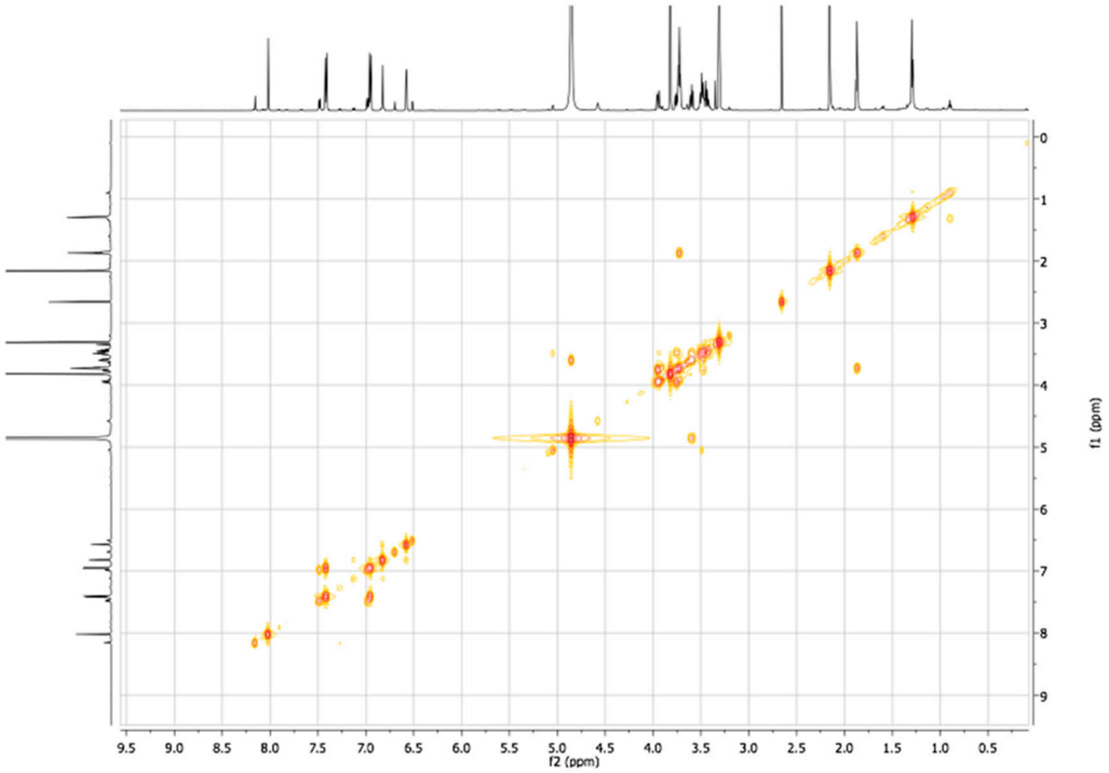

Figure S3. COSY (150 MHz, CD_3OD) spectra of mixture of sissotrin and isosissotrin obtained by biotransformation by *Absidia coerulea* AM 93.

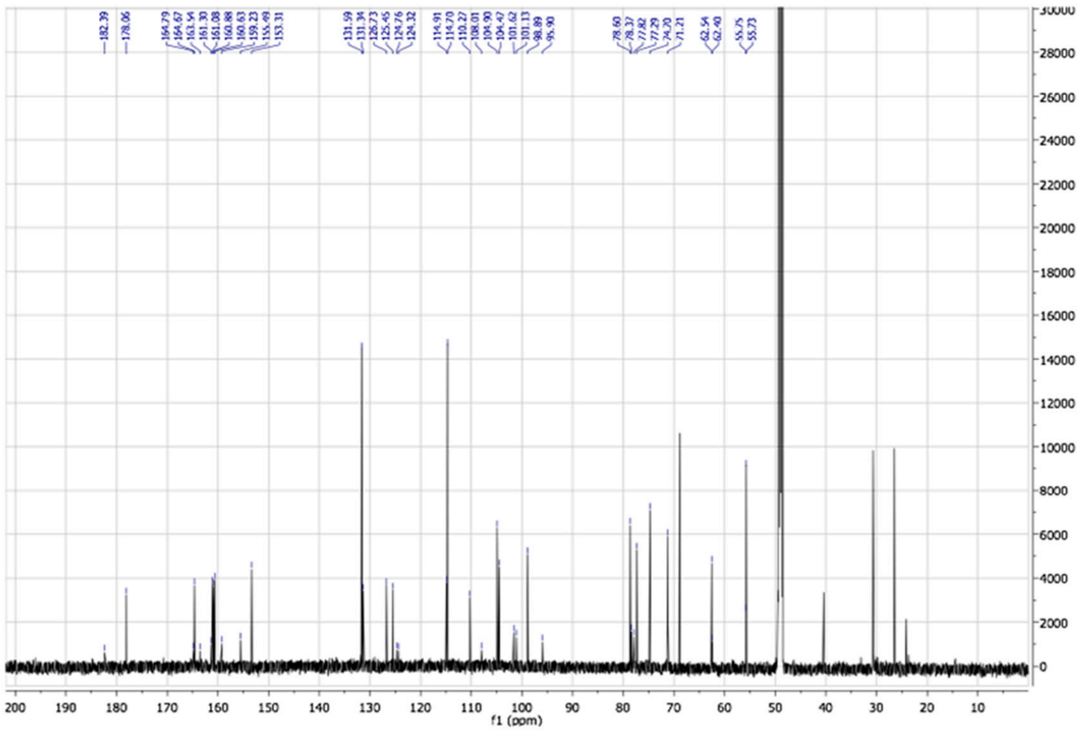

Figure S4. ^{13}C-NMR (150 MHz, CD_3OD) spectra of mixture of sissotrin and isosissotrin obtained by biotransformation by *Absidia coerulea* AM 93.

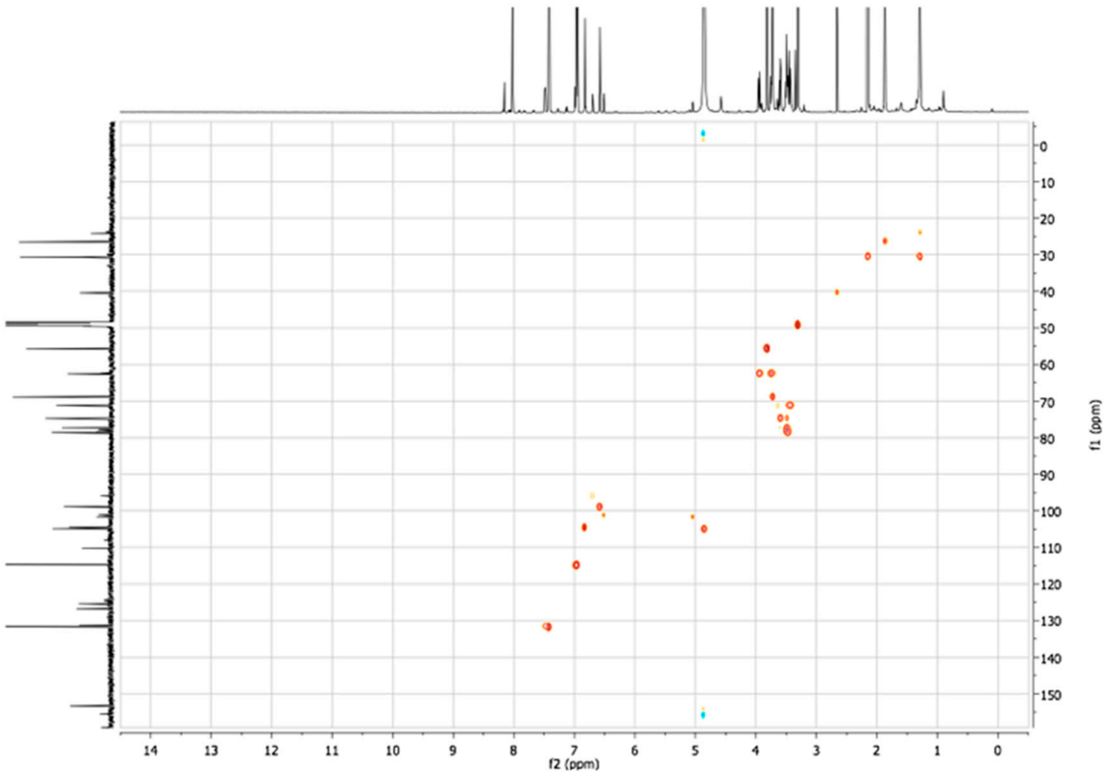

Figure S5. HSQC (150 MHz, CD_3OD) spectra of mixture of sissotrin and isosissotrin obtained by biotransformation by *Absidia coerulea* AM 93.

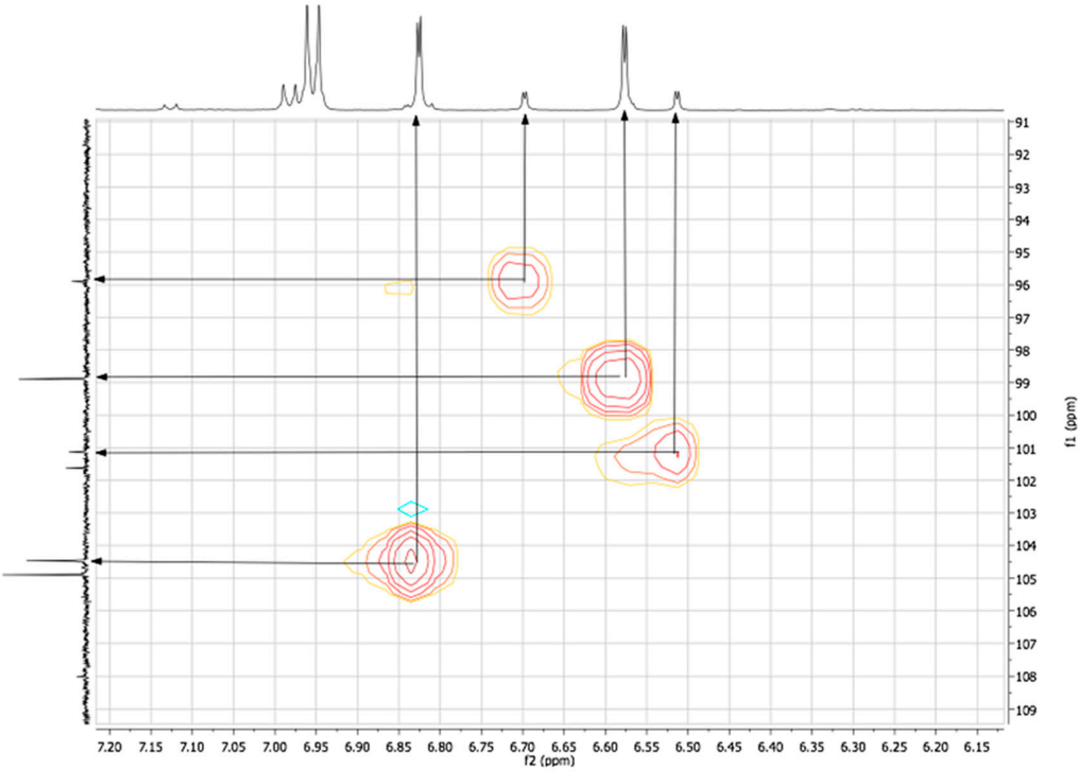

Figure S6. HSQC (150 MHz, CD_3OD) spectra of mixture of sissotrin and isosissotrin (correlations of aromatic A-ring protons: H-6 and H-8) obtained by biotransformation by *Absidia coerulea* AM 93.

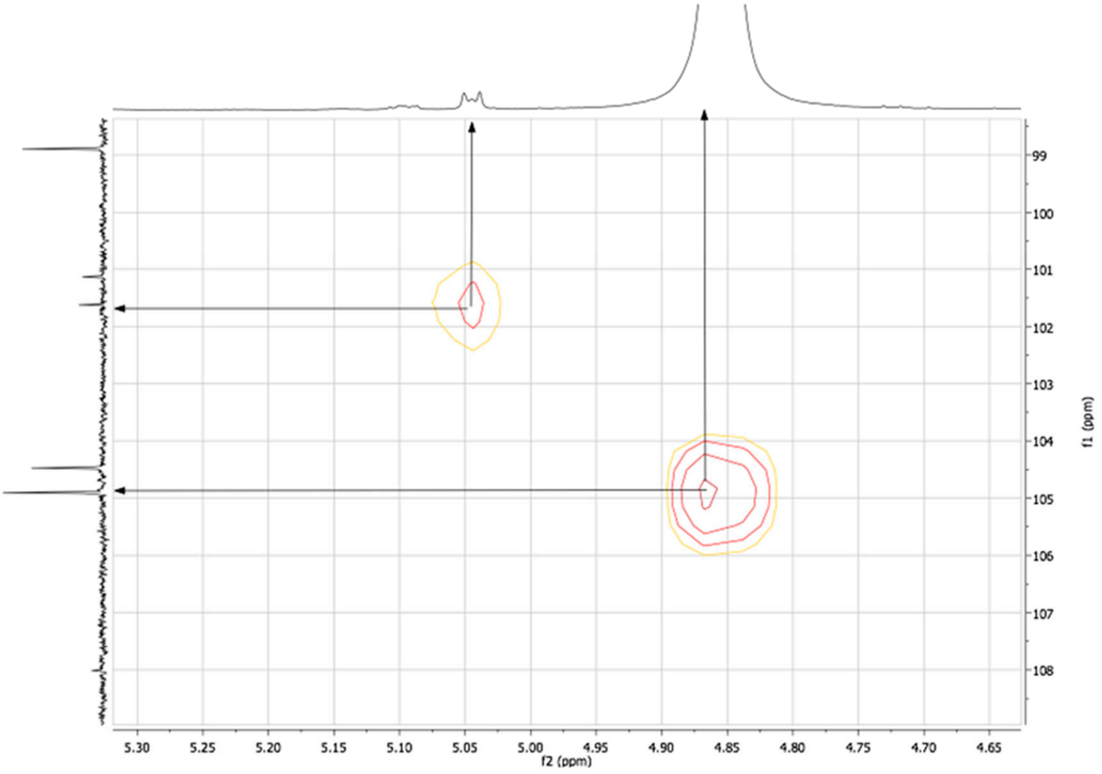

Figure S7. HSQC (150 MHz, CD_3OD) spectra of mixture of sissotrin and isosissotrin (correlations of H-1″ protons) obtained by biotransformation by *Absidia coerulea* AM 93.

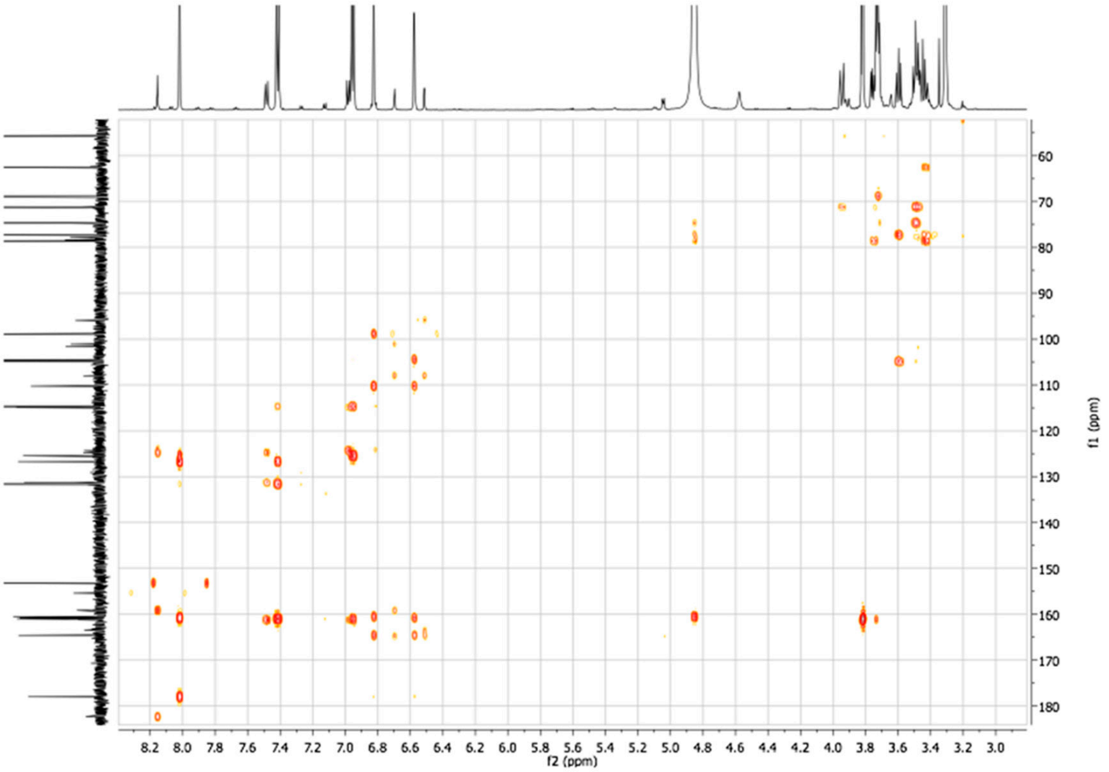

Figure S8. HMBC (150 MHz, CD_3OD) spectra of mixture of sissotrin and isosissotrin obtained by biotransformation by *Absidia coerulea* AM 93.

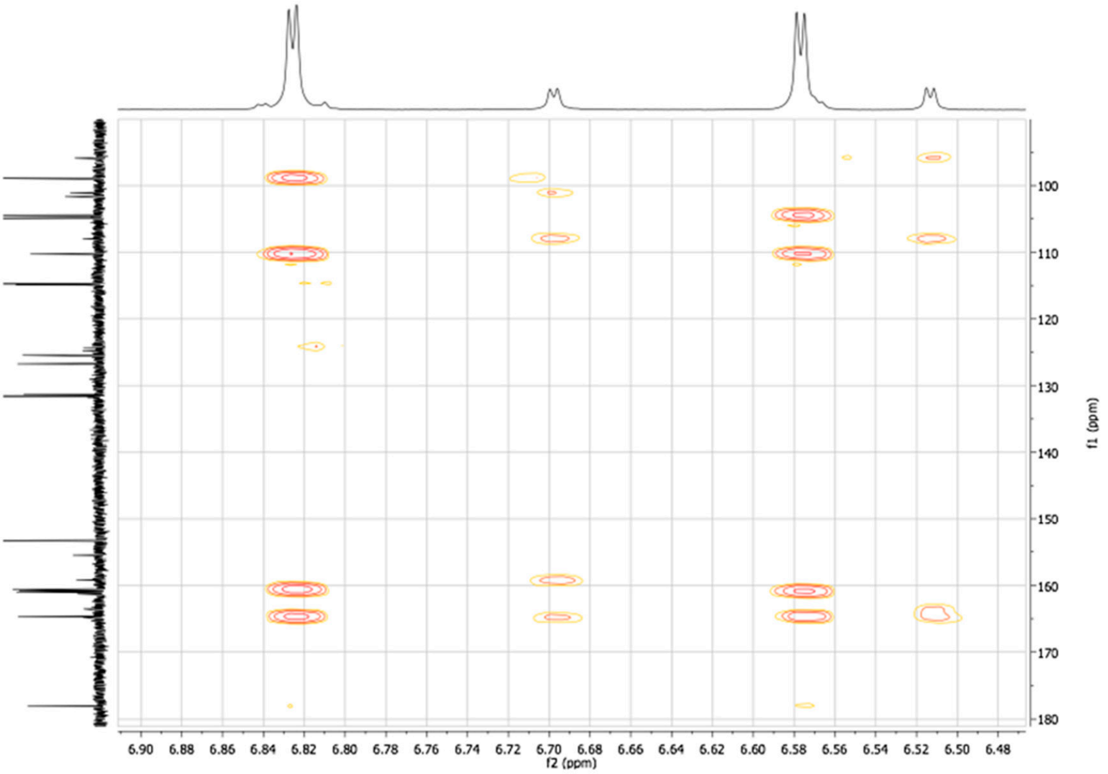

Figure S9. HMBC (150 MHz, CD_3OD) spectra of mixture of sissotrin and isosissotrin (correlations of aromatic A-ring protons-H-6 and H-8) obtained by biotransformation by *Absidia coerulea* AM 93.

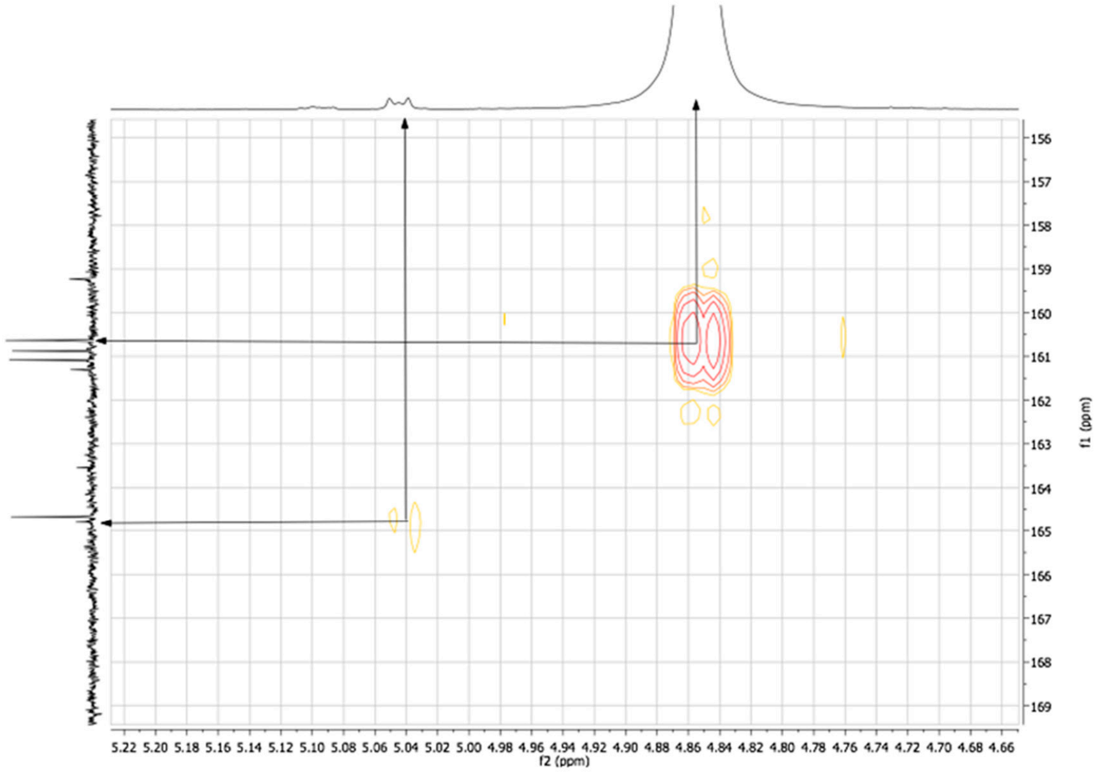

Figure S10. HMBC (150 MHz, CD3OD) spectra of mixture of sissotrin and isosissotrin (correlations of H-1″ protons) obtained by biotransformation by *Absidia coerulea* AM 93.

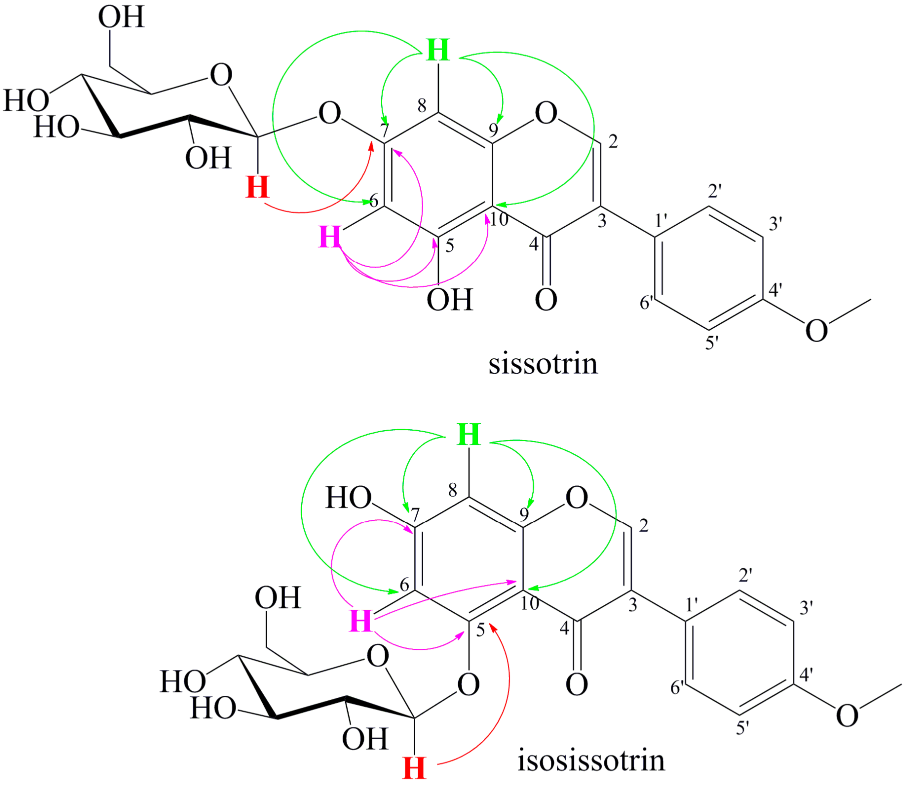

Figure S11. HMBC correlations for sissotrin and isosissotrin obtained by biotransformation by *Absidia coerulea* AM 93.

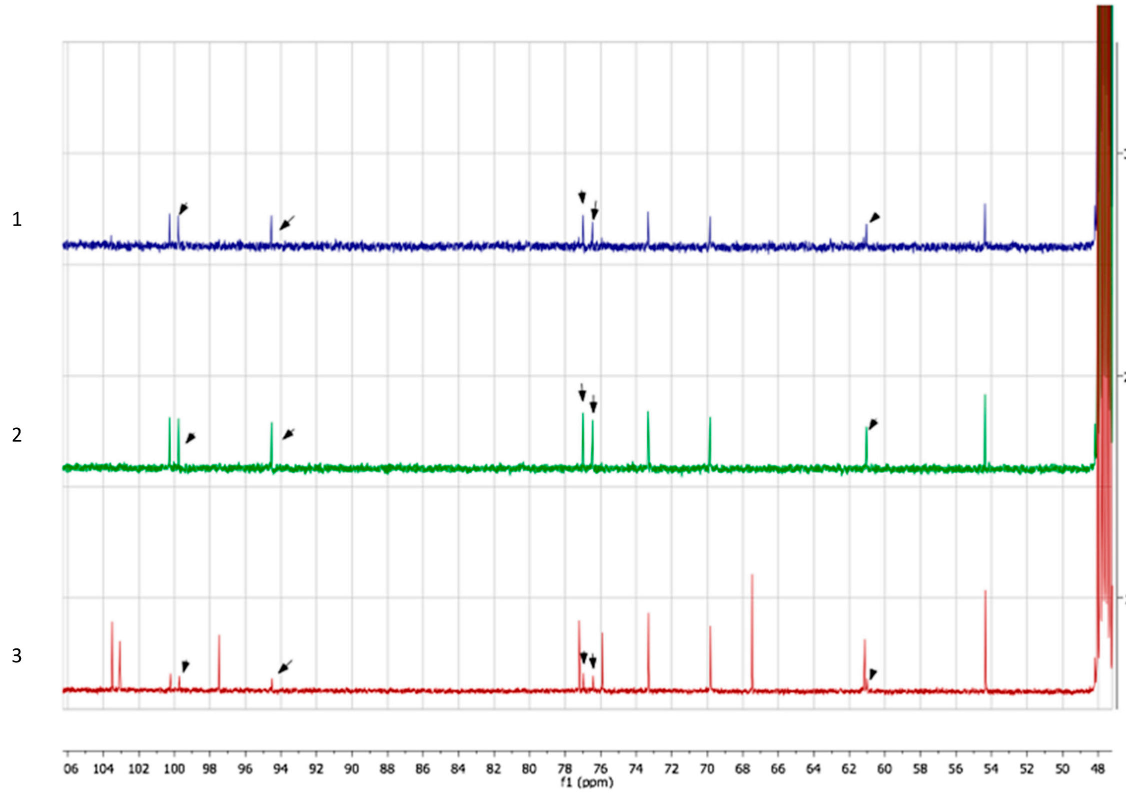

Figure S12. ^{13}C-NMR (150 MHz, CD_3OD) spectra of sissotrin (obtained by biotransformation by *Absidia glauca* AM 177 (1) and by *Rhizopus nigricans* UPF 701 * (2)) and mixture of sissotrin and isosissotrin obtained by biotransformation by *Absidia coerulea* AM 93 (3). Arrows indicated selected carbon signals of sissotrin. On the NMR spectra of products obtained by biotransformation by *Absidia coerulea* AM 93 those carbon signals are doubled. * Result not included in this paper.

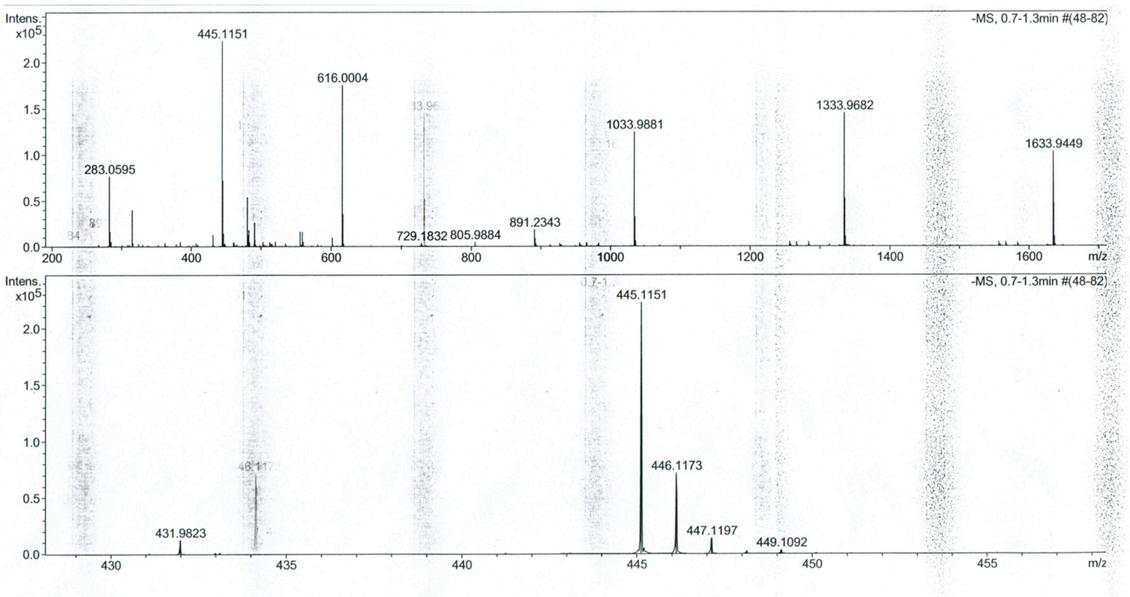

Figure S13. Negative-ion HR-ESIMS spectra of mixture of sissotrin and isosissotrin obtained by biotransformation by *Absidia coerulea* AM 93.
